# Sex Differences and the Influence of an Active Lifestyle on Adiposity in Patients with McArdle Disease

**DOI:** 10.3390/ijerph17124334

**Published:** 2020-06-17

**Authors:** Irene Rodríguez-Gómez, Alfredo Santalla, Jorge Diez-Bermejo, Diego Munguía-Izquierdo, Luis M. Alegre, Gisela Nogales-Gadea, Joaquín Arenas, Miguel A. Martín, Alejandro Lucia, Ignacio Ara

**Affiliations:** 1 GENUD Toledo Research Group, Universidad de Castilla-La Mancha, 45071 Toledo, Spain; irene.rodriguez@uclm.es (I.R.-G.); Luis.Alegre@uclm.es (L.M.A.); 2 CIBER of Frailty and Healthy Aging (CIBERFES), 28029 Madrid, Spain; asanher@upo.es (A.S.); dmunizq@upo.es (D.M.-I.); alejandro.lucia@universidadeuropea.es (A.L.); 3 Department of Sport and Computer Science, Section of Physical Education and Sports, Faculty of Sport, Universidad Pablo de Olavide, 41013 Sevilla, Spain; 4 Research Institute Hospital 12 de Octubre, 28041 Madrid, Spain; jorge.cafyd@gmail.com (J.D.-B.); joaquin.arenas@salud.madrid.org (J.A.); mamcasanueva@h12o.es (M.A.M.); 5 Department of Neurosciences, Institut d’Investigació en Ciències de la Salut Germans Trias i Pujol I Campus Can Ruti, Universitat Autònoma de Barcelona, 08041 Badalona, Spain; gnogales@igtp.cat; 6 CIBER of Rare Disorders (CIBERER), 28029 Madrid, Spain; 7 School of Research and Doctorate Studies, Universidad Europea de Madrid, 28670 Madrid, Spain

**Keywords:** DXA, fat mass, exercise, physical activity, inactivity, obesity

## Abstract

McArdle disease (glycogenosis-V) is associated with exercise intolerance, however, how it affects an important marker of cardiometabolic health as it is adiposity remains unknown. We evaluated the association between physical activity (PA) and adiposity in patients with McArdle disease. We assessed 199 adults of both sexes (51 McArdle patients (36 ± 11 years) and 148 healthy controls (35 ± 10 years)). Body fat (BF) was determined using dual-energy X-ray absorptiometry (DXA) method and each patient’s PA was assessed with the International PA Questionnaire (IPAQ). Although body mass index values did not differ between patients and controls, McArdle patients had significantly higher values of BF in all body regions (*p <* 0.05) and higher risk of suffering obesity (odds ratio (OR): 2.54, 95% confidence interval (95% CI): 1.32–4.88). Male patients had higher BF and obesity risk (OR: 3.69, 95% CI: 1.46−9.34) than their sex-matched controls, but no differences were found within the female sex (*p <* 0.05). In turn, active female patients had lower trunk BF than their inactive peers (*p <* 0.05). Males with McArdle seem to have adiposity problems and a higher risk of developing obesity than people without the condition, while female patients show similar or even better levels in the trunk region with an active lifestyle. Therefore, special attention should be given to decrease adiposity and reduce obesity risk in males with McArdle disease.

## 1. Introduction

McArdle disease, also known as glycogenosis type V, is a myopathy caused by inherited deficiency of the muscle isoform of glycogen phosphorylase, *myophosphorylase* [1,2]. The activity of this enzyme is typically absent with a subsequent blockade of muscle glycogen breakdown [3]. Affected individuals present with exercise intolerance in the form of acute crises of fatigue and muscle stiffness and contractures, especially at the start of exercise, which is usually attenuated if exercise is stopped or intensity is reduced [4]. These episodes can be accompanied by rhabdomyolysis, as reflected by abrupt elevations in serum creatine kinase (CK) levels (a marker of muscle damage) and myoglobinuria (‘dark urine’) [5]. As a result, physical activity (PA) has traditionally been contraindicated in these patients by clinicians [6,7,8,9,10] to the extent that some authors have suggested that clinicians should encourage patients to adopt an active rather than a sedentary lifestyle [6,7,11]. This inactivity, in addition to not improving the clinical condition or attenuating the progression of muscle damage [10,12], exacerbates the exercise intolerance of these patients [11,12,13] and results in a deterioration of physical capacity with a consequent impairment of quality of life [10,11,12,14]. In turn, exercise and PA seems to be the main modifier of the clinical course of McArdle disease [7,9]. In this context, previous research has shown that regular, light/moderate-intensity aerobic exercise interventions (e.g., brisk walking), improve the physical capacity (expressed as cardiorespiratory fitness (CRF)) of patients with McArdle disease [15,16,17,18], and benefits have also been reported in this patient population with a supervised resistance exercise intervention [19]. 

In relation to the body composition, inactivity and low CRF are associated with a higher prevalence of obesity [20,21] and with a higher risk of all-cause mortality [22,23,24,25]. In contrast, higher levels of PA and/or CRF reduce the risk of premature death [24,26] and improve body composition, as well as decreasing and increasing body fat (BF) and lean mass, respectively [27]. In McArdle patients, higher PA levels are associated with a healthier body composition phenotype (i.e., higher lean and bone mass), with active patients showing higher lean mass than their sedentary patient peers [28]. Thus, inactivity might likely lower the daily energy expenditure of these patients, potentially leading to an increase in adiposity and thus to a decrease in patients’ health status. However, whether the levels of PA can actually influence the adiposity accumulation and distribution has not been previously studied in patients with McArdle disease.

The purpose of our study was to compare the BF profile of McArdle patients versus age/sex-matched controls and to determine the effect of PA on the patients’ adiposity indices. In addition, we sought to determine the risk factor for obesity in McArdle patients and especially, in inactive patients. Our hypothesis was that BF would be higher in both male and female patients with McArdle disease than in healthy individuals, and that an active lifestyle could improve this situation and reduce the risk of obesity.

## 2. Materials and Methods

### 2.1. Patients

**Figure 1:**
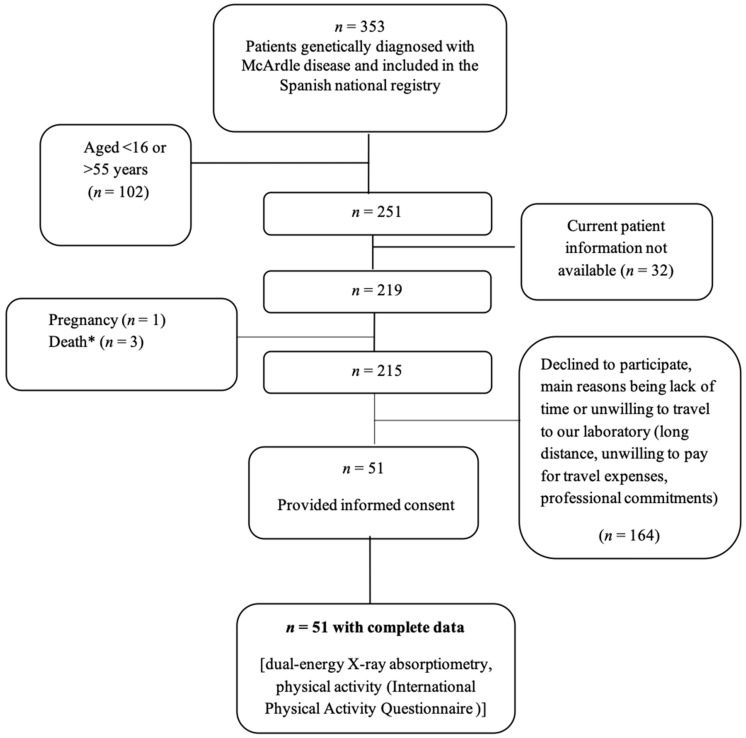
Flow diagram of patients with McArdle disease. * Death due to causes independent of McArdle disease (i.e., cardiovascular disease).

The study protocol was approved by the ethics committee of the Research Institute of the Hospital 12 de Octubre (Madrid, Spain; reference #16/081) and adhered to the tenets of the Declaration of Helsinki 1961 (revision Edinburgh 2000). Patients were recruited for this study if they met the following criteria: (i) genetic diagnosis of McArdle disease, that is, identification of the two mutant alleles in the gene (*PYGM*) encoding myophosphorylase, as determined elsewhere [13] or, in those in whom only one mutant allele has been identified to date, biopsy diagnosis or alternatively laboratory confirmation of the ‘pathognomonic’ second wind phenomenon [12]; (ii) age 16 to 55 years; and (iii) having no condition contraindicating dual-energy X-ray absorptiometry (DXA; e.g., pregnancy). A total of 51 patients with McArdle disease (26 males, 25 females) who met all inclusion criteria and provided informed consent were evaluated during June 2015–May 2019 (Figure 1). All the patients were informed of the aims and procedures of the study, as well as of the possible risks and benefits.

### 2.2. Controls

Age- and gender-matched healthy subjects with previous DXA data collected by us using the same equipment (see below) during the 2012–2019 period were used at as patient-controls with a ratio as close as possible to ~1:3. Of the 849 possible subjects between 16 and 55 years, 168 subjects with previous comorbidities were excluded from the sample (i.e., diabetes, cancer, etc.). Finally, we had 365 healthy male and 316 healthy females before matched. A total of 148 healthy subjects were contacted again by us and agreed to serve as controls for the current study. Subjects written informed consent was obtained from all of them. The following outcomes (see below) were recorded.

### 2.3. Anthropometry 

Anthropometric measurements were obtained from all the participants immediately before DXA assessment. Height was measured in the upright position, in underwear and barefoot on a stadiometer with a precision of 1 mm (Seca 711; Hamburg, Germany). Body mass (BM) was determined with the same requirements using a balance with a 100 g precision (Seca 711). Body mass index (BMI) was calculated as body mass divided by height squared (kg/m^2^).

### 2.4. Adiposity

Total and regional BF were assessed in all the study participants using the same DXA equipment (Hologic QDR Discovery; Bedford, MA, United States). DXA equipment was calibrated using a lumbar spine phantom and following the Hologic guidelines. All DXA scan tests were analyzed using Physician’s Viewer, APEX System Software Version 3.1.2. (Hologic QDR Discovery; Bedford, MA, United States). BF (kg) was calculated from total and regional analysis of the whole-body scan. With this analysis, regional BF can be assessed with a coefficient of variation below 5% [29]. Scans were made with subjects in a supine position, wearing light clothing with no metal and no shoes or jewelry. To classify our sample into the obesity subgroup, we used the reference values established by Lohman et al., with cut-off points at 25% BF in male and 35% BF in female [30].

### 2.5. Physical Activity in Patients

The Spanish long-form version of the International Physical Activity Questionnaire (IPAQ) was used to assess the PA habits of the patients. This questionnaire records information on the frequency, intensity, and duration of occupational, transport, home, and leisure/sport activities performed in the previous seven days. It has been validated against accelerometry and is widely used to evaluate the patterns of PA at the international level [31,32]; moreover, it has shown to have satisfactory psychometric properties [31,33]. The IPAQ is also suitable for assessing PA in patient populations [31] as it is divided in different parts, each addressing the specific types of PA that patients with chronic disease are most likely to do [34]. This questionnaire has been used previously to assess the PA levels of Spanish patients with McArdle disease [14,28]. 

We categorized participants into two groups according to their leisure-time PA levels: (i) “active”, if they completed ≥600 metabolic equivalents (MET)·min·week^−1^, where 1 MET is the resting energy expenditure, equivalent to an oxygen consumption of ~3.5 mL·kg^−1^·min^−1^ and 600 MET·min·week^−1^ corresponds to the minimum level of moderate–vigorous PA (150 min/week) recommended by the World Health Organization for all adults [35]; or (ii) “inactive” if their PA levels were below 600 MET·min·week^−1^ [14,36]. The methods used to score the long IPAQ can be found at the IPAQ Web site (www.ipaq.ki.se).

### 2.6. Statistical Analysis

Statistical analyses were performed with the IBM SPSS statistics package version 25 (SPSS, Inc.; Chicago, IL, USA). The Kolmogorov–Smirnov test and graphical methods (normal probability plots) were used to determine the normal distribution of the variables. Descriptive statistics were run on age and anthropometric variables. Bivariate analysis was conducted using the chi-square test between patients and controls, and active and inactive patients, both for the whole group and also separately by sex. Odds ratios (OR) and their respective 95% confidence intervals (95% CI) were also calculated. An analysis of covariance (ANCOVA) was used to determine if there existed differences in adiposity indices (i.e., total/regional fat mass) for the comparison of (i) patients versus controls (as a whole and also separately by sex), and of (ii) active versus inactive patients (within each sex). In all ANCOVA analyses age, body mass, height, age of symptom onset, and frequency of rhabdomyolysis episodes were used as covariates, and we applied the Bonferroni post-hoc test. Also, the continuous association between time spent in moderate, vigorous, and leisure activities with the outcomes related to BF in the study were explored via linear regression in males and females separately. The same set of covariates used for the ANCOVA analysis was used. The statistical effect size (ES) was reported with the Hedge’s g test for comparisons of unequal sample size. The level of statistical significance was set at *p* ≤ 0.05. 

## 3. Results

A total of 51 patients (26 males, 25 females; aged 16 to 55 years) and 148 controls (76 males, 72 females) participated in the study (Table 1). 

Table 2 shows main demographic data for the participants by group and sex. Patients and controls as a whole group and divided by sex did not differ significantly in age, BM, or BMI adiposity, with the exception of height, which was significantly higher in male controls than male patients (*p <* 0.05). The results of total and regional BF values by group and sex are shown in Table 3. 

McArdle patients as a whole group (male and female together) showed significantly higher values of whole-body and regional BF than their controls (between-group differences ranging from 17.5% to 21.4%, all *p <* 0.05). The ES values for these differences were large for the trunk (Hedge’s g = 0.86) and medium for the rest of indices (Hedge’s g averaging 0.60). Likewise, when male patients were compared with their sex-matched controls, all adiposity indices showed a similar trend, that is, greater values (by 22% on average) in the former (Hedge’s g ranging from 0.01 to 1.02, all *p <* 0.05). In contrast, no differences were found between female subgroups. The results of total and regional BF values in patients based on PA levels are shown in Table 4. Nevertheless, no significant associations were found when the continuous associations between time spent in moderate, vigorous, and leisure activities with BF were studied.

The comparison between active and inactive McArdle patients distinguishing by sex revealed significant differences only in trunk BF, with higher values in the female subgroup (by 9.7%, with a large ES (Hedge’s g = 0.92; *p <* 0.05)). Similarly, the associations with obesity are shown in Table 5. McArdle patients were significantly more likely to develop obesity than the control group (OR: 2.54, 95% CI: 1.32–4.88). Differentiating by sex, the male patients also showed a higher risk increase for obesity than their counterparts (OR: 3.69, 95% CI: 1.46−9.34), but not the female group. Not significantly increased risk was found in inactive patients. 

Finally, Table 6 summarizes data for the McArdle patient group in relation to the PA. There were significant differences between male and female patients in “domestic activity” (*p <* 0.05). 

## 4. Discussion

This study examines the effects of McArdle disease on adiposity, especially in relation to the sex and patient´s lifestyle (i.e., PA). Our main finding was that although McArdle patients showed similar BMI values than controls, BF values were higher in the former compared to age- and sex-matched controls. However, when groups were divided by sex, this difference with their counterparts was only shown in males. In fact, this case-control study could confirm the association of McArdle disease with obesity, increasing the risk particularly in male patients by almost four-fold. Moreover, it seems that only female patients with an active lifestyle could reduce this adiposity in the trunk region, showing lower BF than the inactive female patients. 

According to our research, despite no differences in BMI values, both the whole-body and the regional BF values in the McArdle group were significantly higher compared to age- and sex-matched control subjects. Interestingly, although the patients were within the normal-weight category, the control group was categorized as overweight or pre-obese according to the BMI classification [37]. The latter might be due to the well-known limitations of the use of BMI for assessing actual adiposity [38]. However, when analyzing %BF, we found the opposite classification, with the McArdle patients being considered as overweight or pre-obese [30]. Therefore, our results again reinforce the importance of using the %BF against BMI when referring to adiposity, also in people with McArdle disease. In addition, the risk of developing obesity was significantly increased in McArdle patients, confirming the positive association of McArdle disease with obesity. This BF accumulation could be due to the fact that McArdle disease has always been characterized by physical inactivity. Consequently, a sedentary lifestyle may increase the accumulation of BF during growth, leading to a higher percentage of BF in adult life. In relation to other glycogenosis such as Pompe disease or type II glycogenosis, some discrepancies have been found. Adult patients with Pompe disease showed higher %BF (39.4%) in one of the studies [39] yet lower %BF (20.7%) in another report [40]. Our results are more comparable to those found by Papadimas et al. [39], because these authors used DXA instruments, whereas Ravaglia et al. used anthropometry and bioelectrical impedance analysis to determine body composition [40]. Therefore, although it is difficult to compare the two conditions (i.e., McArdle vs. Pompe), both are usually characterized by a reduced level of PA and exercise intolerance; thus, their body composition could differ from that of disease-free individuals [39,40]. Despite both diseases showing similar mean BMI results, below the overweight category (24.7 kg/m^2^ in McArdle vs. 23.7 kg/m^2^ in Pompe), the BF accumulation found in McArdle disease could be a condition that develops with age. Indeed, a recent study found no differences in the BF of children and adolescents with McArdle disease compared with their counterparts in spite of the fact that some differences in lean and bone mass were found [41]. 

Both male and female patients showed similar BMI classification and, according to their %BF, would be classified as pre-obese [30]. Nonetheless, when comparing both male and female patients with their corresponding control subgroups, we found that only in the case of male there were significant differences in all regions of the body between patients and controls. Female patients showed similar values compared to their counterparts in all body regions. Furthermore, both female controls and patients would be categorized with the same status (i.e., pre-obese), but control males were normal weight, which is in contrast with male McArdle patients, who were pre-obese/obese. This could be one reason for the female subgroup’s similarity, given that male and female controls had a different status, with females probably being less healthy than males. In addition, another reason could be the PA, as significantly more time was spent in domestic activities by the females with McArdle disease. In the same line, only male patients demonstrated an increased risk for obesity, showing the double prevalence of obese subjects in the patient group (61.5% vs. 30.3%). Thus, the risk of suffering obesity is almost four-fold higher in males with McArdle disease compared with control males. For these reasons, it seems that male patients have a problem of adiposity, which would be especially relevant because of the most dangerous BF accumulation (i.e., visceral). BF accumulation in males did predominate in the abdominal area, indeed, which represents a risk factor per se for other pathologies such as diabetes, hypertension, metabolic syndrome, or cardiovascular diseases [42,43]. Nevertheless, the PA levels of our active male patients might not be enough to improve adiposity indices; since, as can be seen from the Table 4, 50% of the active males had obesity against the 85.7% of the inactive patients. In fact, in general, active patients were not significantly less likely to develop obesity, and no associations were found between PA and BF. Whereas, it is true that in the case of female patients, there seemed to be a significant benefit of PA, at least for the trunk area. Previous studies have shown the essential role of PA in this clinical manifestation of the disease, with an active lifestyle alleviating, at least partly, the consequences of a sedentary lifestyle with regard to body composition (lean and bone mass) and quality of life [28]. The aforementioned differences between sexes could be explained by the domestic PA, given that female patients spent significantly more time in this type of activities than male patients. However, more knowledge is needed to understand the relationship between fat mass and physical activity in McArdle patients.

Finally, when compared with other glycogenosis patients by sex, some discrepancies have been found with the values previously reported in adult patients with Pompe disease. Ravaglia et al. found a lower %BF in Pompe patients than in our McArdle patients for both sexes (male: 18.5% vs. 25.0% here; female: 24.2% vs. 32.9%) [40], while a higher %BF was found by Papadimas et al. (male: 32.0% vs. 25.0% here; female: 46.8% vs. 32.9%) [39]. 

## 5. Limitations and Strengths

This study is not without limitations. Particularly, PA was self-reported and not objectively assessed (e.g., using accelerometry). Nevertheless, we used a validated questionnaire with satisfactory psychometric properties [31,32,33], which in fact has been previously used in this population [14,28]. Related to the control group, we did not have PA information, although eligible subjects engaged in elite sports were excluded from the sample. Furthermore, we did not assess an important nutritional data. In turn, this is the first study that evaluates adiposity in adult patients with McArdle disease, thereby providing information on an important health indicator as is BF. Further, the latter was assessed with the gold standard method, DXA. Of note is also the fact that we assessed a large sample of patients (at least within the context of a rare condition as is McArdle disease).

## 6. Conclusions

Although McArdle patients showed similar BMI compared to age- and sex-matched controls, BF was higher in the former. As for within-sex comparisons, female patients showed similar adiposity levels than their age-matched referents, whereas male patients tended to have higher levels of adiposity in all the body areas than male controls. Therefore, this case-control study may confirm the increased risk to develop obesity in McArdle patients, in particular in the case of male patients, who were had a four-fold increased risk than controls. In addition, it seems that only female patients with an active lifestyle could reduce adiposity in the trunk region, showing lower fat accumulation than the inactive female patients. In this regard, special attention should be given to male patients owing to their tendency for abdominal fat accumulation, which is marker of poor cardiometabolic health. Future studies should further assess whether PA interventions can improve the adiposity profile of patients, particularly in men. 

## Figures and Tables

**Table 1:** *PYGM* mutations identified in the study patients (*n* = 51).

Type of Mutation	*n*	%
p.R50X (c.148C>T)/p.R50X (c.148C>T)	22	43.14
p.R50X (c.148C>T)/p.G205S (c.613G>A)	6	11.76
p.R50X (c.148C>T)/p.W798R (c.2392T>C)	5	9.80
p.R50X (c.148C>T)/p.L5VfsX22 (c.13_14delCT)	2	3.92
p.R50X (c.148C>T)/p.Q755X (c.2263C>T)	2	3.92
p.G205S (c.613G>A)/c.1768 + 1G>A	2	3.92
p.G205S (c.613G>A)/p.G205S (c.613G>A)	1	1.96
p.R50X (c.148C>T)/c.1768 + 1G>A	1	1.96
p.R50X (c.148C>T)/p.R194W (c.580C>T)	1	1.96
p.R50X (c.148C>T)/p.V456M (c.1366G>A)	1	1.96
p.R194W (c.580C>T) + p.E797VfsX18 (c.2385_2386delAA)/p.R194W (c.580C>T) + p.E797VfsX18 (c.2385_2386delAA)	1	1.96
p.W388SfsX34 (c.1162_1169delTGGCCGGT)/p.W388SfsX34 (c.1162_1169delTGGCCGGT)	1	1.96
p.K754NfsX49 (c.2262delA)/p.K754NfsX49 (c.2262delA)	1	1.96
p.R771PfsX33 (c.2310_2311dupCC)/p.R771PfsX33 (c.2310_2311dupCC)	1	1.96
p.W798R (c.2392T>C)/c.212_218dup (p.Q73HfsX7)	1	1.96
p.L5VfsX22 (c.13_14delCT)/p.K754Nfsx49 (c.2262delA)		1.96
p.Q734HfsX7 (c.211_217dupCGCAGCA)/p.Q734HfsX7 (c.211_218dupCGCAGCA)	1	1.96
c.244−3_244−2delCA/c.1093−1G>T	1	1.96

**Table 2:** Anthropometric and descriptive data by group and sex.

	Patients (*n* = 51)			Controls (*n* = 148)			Male Patients (*n* = 26)			Male Controls (*n* = 76)			Female Patients (*n* = 25)			Female Controls (*n* = 72)		
Sex (%)																		
Male	51			51														
Female	49			49														
Age (years)	36	±	11	35	±	10	33.6	±	11.8	33.4	±	11.4	38.0	±	8.8	37.3	±	8.5
BM (kg)	72.0	±	15.4	70.5	±	13.6	76.1	±	14.7	77.5	±	9.5	67.7	±	15.3	63.1	±	13.4
Height (cm)	170.5	±	7.1	169.9	±	13.4	174.2	±	5.2 *	177.4	±	5.7	166.6	±	6.8	161.9	±	14.5
BMI (kg/m^2^)	24.7	±	5.0	27.2	±	40.3	25.0	±	4.2	24.7	±	3.1	24.5	±	5.8	30.0	±	57.7

Data are mean ± standard deviation. Abbreviations: BM, body mass; BMI. body mass index. * *p* < 0.05 for male patients vs. male controls.

**Table 3:** Adiposity (fat mass) indices by group and sex.

							Fat Mass (kg)											
	Patients (*n* = 51)			Controls (*n* = 148)			Male Patients (*n* = 26)			Male Controls (*n* = 76)			Female Patients (*n* = 25)			Female Controls (*n* = 72)		
Whole-body	21.4	±	0.8	17.5	±	0.5 *	19.9	±	0.8	15.6	±	0.5 †	21.2	±	0.8	20.2	±	20.2
Subtotal body	20.6	±	0.8	16.7	±	0.5 *	19.0	±	0.8	14.6	±	0.5 †	20.4	±	0.8	19.4	±	19.4
Trunk	10.3	±	0.4	8.1	±	0.2 *	10.3	±	0.4	7.8	±	0.3 †	9.4	±	0.5	8.8	±	8.8
Arms (mean)	1.1	±	0.1	0.9	±	0.0 *	1.0	±	0.0	0.8	±	0.0 †	1.1	±	0.1	1.1	±	1.1
Legs (mean)	4.0	±	0.2	3.3	±	0.1 *	3.3	±	0.2	2.6	±	0.1 †	4.4	±	0.2	4.2	±	4.2
% BF	29.6	±	1.0	25.3	±	0.6 *	25.0	±	0.8	19.7	±	0.6 †	32.9	±	0.9	31.7	±	0.5

Data are mean ± standard error of the mean; a. * *p* < 0.05 for patients vs. controls; b. † *p* < 0.05 for male patients vs. male controls.

**Table 4:** Fat mass indices in McArdle patients by physical activity levels and sex.

Fat Mass (kg)												
	Active Male Patients (*n* = 18)			Inactive Male Patients (*n* = 7)			Active Female Patients (*n* = 16)			Inactive Female Patients (*n* = 9)		
Whole-body	19.8	±	0.6	20.5	±	1.1	23.3	±	0.5	24.0	±	0.7
Subtotal body	18.9	±	0.6	19.6	±	1.1	22.5	±	0.5	23.2	±	0.7
Trunk	10.3	±	0.3	10.9	±	0.6	10.3	±	0.3	11.3	±	0.4 *
Arms (mean)	1.0	±	0.1	1.1	±	0.1	1.2	±	0.1	1.3	±	0.1
Legs (mean)	3.3	±	0.2	3.3	±	0.3	4.9	±	0.2	4.7	±	0.3

Data are mean ± standard error of the mean; * *p* = 0.05 for active female patients vs. inactive female patients.

**Table 5:** Association between McArdle disease and obesity (**a**), and physical activity and obesity only in McArdle patients (**b**).

		*n*	*n* Obesity	% Obesity	OR	95% CI			*p*-Value
**(a)**		**Disease**							
Whole Sample									
	Patients	51	26	51.0%	2.54	(1.32–4.88)			0.005 *
	Controls	148	43	29.1%					
Male									
	Patients	26	16	61.5%	3.69	(1.46–9.34)			0.005 *
	Controls	76	23	30.3%					
Female									
	Patients	25	10	40.0%	1.73	(0.67–4.49)			0.255
	Controls	72	20	27.8%					
**(b)**		**Physical Activity in McArdle Patients**							
Whole Sample									
	Active	34	16	47.1%	0.69	(0.21–2.29)			0.544
	Inactive	16	9	56.3%					
Male									
	Active	18	9	50.0%	0.17	(0.02–1.68)			0.102
	Inactive	7	6	85.7%					
Female									
	Active	16	6	40.0%	1.00	(0.20–5.12)			1.000
	Inactive	9	4	40.0%					

Abbreviations: CI, confidence interval; OR, odds ratio; * *p* < 0.05 for patients vs. controls.

**Table 6:** Physical Activity categories adjusted by age.

	Activity (MET·min·week^−1^)								
Activity	Patients (*n* = 51)			Male Patients (*n* = 26)			Female Patients (*n* = 25)		
Walk	1509	±	272	1574	±	390	1445	±	390
Moderate	3362	±	465	3276	±	666	3449	±	666
Vigorous	1401	±	441	1413	±	632	1389	±	632
Work	2395	±	656	2724	±	940	2066	±	940
Transport	574	±	87	638	±	125	511	±	125
Domestic	1631	±	211	1165	±	303	2097	±	303 *
Leisure time	1733	±	290	1842	±	415	1623	±	415

Data are mean ± standard deviation; Abbreviations: MET, metabolic equivalent; * *p* < 0.05 for male vs. female patients.
